# The effect of cardiac rehabilitation on anxiety and depression in patients undergoing cardiac bypass graft surgery in Iran

**DOI:** 10.1186/1471-2261-12-40

**Published:** 2012-06-08

**Authors:** Farkhondeh Sharif, Alireza Shoul, Mansour Janati, Javad Kojuri, Najaf Zare

**Affiliations:** 1 Mental Health Nursing Department, Community Based Nursing & Midwifery Research Center, Faculty of Nursing and Midwifery, Shiraz University of Medical Sciences, Shiraz, Iran; 2 Faghihi Hospital, Cardiac Surgery Unit, Shiraz University of Medical Sciences, Shiraz, Iran; 3 Department of Surgery, Shiraz University of Medical Sciences, Shiraz, Iran; 4 Department of Internal Medicine, Shiraz University of Medical Sciences, Shiraz, Iran; 5 Department of Biostatistics, Shiraz University of Medical Sciences, Shiraz, Iran; 6 Department of Psychiatric& Mental Health Nursing, Shiraz University of Medical Sciences, Shiraz, Iran

**Keywords:** Coronary artery bypass graft, Anxiety, Depression, Rehabilitation

## Abstract

**Background:**

Many patients experience anxiety and depression after cardiac bypass surgery.

The aim of this study was to examine the effect of cardiac rehabilitation on anxiety and depression in patients undergoing coronary artery bypass grafting in hospitals affiliated to Shiraz University of Medical Sciences in southern Iran.

**Methods:**

For this randomized controlled trial, 80 patients who met the inclusion criteria were recruited and randomly assigned to case and control groups. Anxiety was measured with the Spielberger Anxiety Scale and depression was measured using Beck’s Depression Inventory at three points in time: on discharge from the hospital, immediately after the intervention, and 2 months after cardiac rehabilitation. After measuring anxiety and depression in both groups upon discharge, the experimental group participated in 8 cardiac rehabilitation sessions over a 4-week period. The control group received only the routine follow-up care.

**Results:**

There was a statistically significant difference in depression scores between groups at all three time-points (Mean score from 19.6 to 10 in the intervention group and from 19.5 to 14 in the control group, P = 0.0014). However, no significant difference was seen in anxiety scores between the groups (Mean score from 37 to 28 in the intervention group and from 38 to 32 in the control group, P = 0.079).

**Conclusions:**

Cardiac rehabilitation was effective in reducing depression 2 months after surgery in patients undergoing coronary artery bypass grafting.

**Trial registration:**

IRCTN201203262812N8

## Background

Cardiovascular diseases are the most common cause of mortality worldwide, and coronary artery diseases are the most common among all cardiovascular illnesses. They accounted for 50% of deaths in developed countries, and each year many people die due to lack of treatment or suffer from related chronic disabilities [1]. Forty percent of men and 20% of women suffer from cardiovascular diseases. Moreover, health care costs and the costs of medication, equipment, and rehabilitation are estimated to be over 62 billion dollars [2]. Coronary artery disease causes arterial stenosis, compromises blood circulation to the myocardium, and leads to ischemia and infarction because of the build-up of unnatural lipid, fat and fiber in the vessel wall [3,4].

Treatment methods for coronary artery disease consist of angioplasty, drug therapy, stenting, atherectomy, and surgical treatments such as coronary artery bypass graft (CABG) surgery [5]. However, CABG is still the first choice for patients with severe coronary artery disease [6] since drug therapy is incomplete without follow up in cardiac rehabilitation, cardiac rehabilitation, as a secondary prevention, plays an important role among non-pharmacological interventions [7]. In a study by Dadvand et al. (2008) of 66 patients with myocardial infarction, the number of relapses decreased after 8 weeks of cardiac rehabilitation [8].

Coronary artery bypass graft surgery is a traumatic event that causes considerable anxiety and depression for patients. Therefore, one of the main aims of nursing is to enhance their comfort and reduce their stress [9]. In a study, 53 patients who were scheduled for CABG were examined a few days before and after the operation. They completed the Speilberger Anxiety Questionnaire. Patients who had agreed to undergo coronary artery bypass graft surgery experienced considerable anxiety before (55%), shortly after (34%), and 3 months after surgery (32%) [10]. Crowe et al. (1996) reported that symptoms of anxiety are common among patients who are hospitalized for cardiac complications [11]. Signs of depression are seen in 32% of patients before surgery, in 28% immediately after surgery, and in 26% 3 months after surgery [12]. In another study of 155 patients undergoing CABG who were depressed or anxious before surgery, they also had a higher level of depression and anxiety after surgery [13]. Cardiac rehabilitation is a group [14] and interdisciplinary [15] intervention aimed at reducing the risk of further cardiac events and conditions through individual and group training and physical activities. Rehabilitation is used to enhance the patients’ quality of life, encourage them to return to work and their previous life style, improve their social and mental condition, and prevent future cardiac complications [3]. Most studies have shown that rehabilitation programs after surgery have been effective in reducing the amount of depression and anxiety and increasing the quality of life [16-18].

Cardiac rehabilitation describes all measures used to help people with heart disease return to an active and satisfying life to prevent the recurrence of cardiac events. Cardiac rehabilitation services consist of three types or stages: inpatient, outpatient and ongoing prevention. Inpatient rehabilitation begin as soon as possible after admission to hospital, outpatient rehabilitation is after patient discharge from the hospital, and ongoing prevention approaches consist of maintaining the patient’s long term cardiovascular stability and physical condition [3,4,19,20]. In a case–control study, Lin and colleagues (2002) reported reduced anxiety rates on discharge compared to the admission day after the patients had undergone phase 1 (inpatient) cardiac rehabilitation [21].

Since in Iran we do not have sufficient cardiac rehabilitation centers, we started cardiac rehabilitation after CABG surgery. Improvements in technology in developing societies are leading to changes in life style such as reduce of physical activity, change dietary patterns, and thus lead to increases in risk factors for various diseases such as diabetes and coronary artery disease. Because of the increasing numbers of patients, the capacity measures needed for cardiac rehabilitation are not always taken and existing rehabilitation training is insufficient. This study was designed to determine the effect of cardiac rehabilitation on depression and anxiety in patients undergoing CABG.

## Methods

### Design

In this randomized controlled trial, the effects of cardiac rehabilitation on anxiety and depression were investigated in patients after CABG. All patients were evaluated in three stages (before intervention, immediately after intervention, and 2 months after intervention) at Nemazee and Faghihi hospitals in Shiraz, southern Iran. These hospitals are the referral centers for Fars province in southwest Iran.

### Patients

The research population consisted of patients scheduled for CABG. The inclusion criteria were: 40–70 years of age, an ejection fraction ratio of over 40%, scheduled for CABG by a cardiologist, ability to read, write, perform the exercises in the rehabilitation program, and attend rehabilitation sessions.

**Figure 1:**
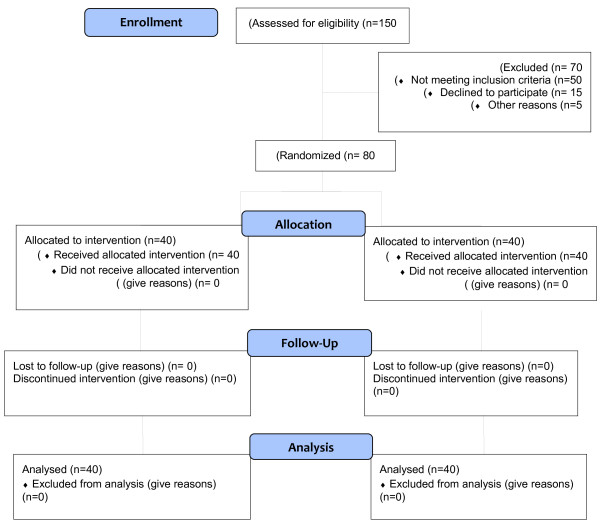
Patients’ flowchart.

The exclusion criteria were: history of mental illness, previous participation in cardiac rehabilitation programs, death or recurrence of illness and hospitalization, disorder or disability that interfered with the patient’s ability to do the rehabilitation exercises, history of cardiac surgery, urgent need for surgery, history of coronary artery transplant along with other operations such as valve replacement, history of severe depression or anxiety and use of antianxiety and antidepressant drugs, history of participating in yoga and meditation programs, severe depression or anxiety, inability to perform exercise and refusal to participate in rehabilitation sessions. The patients’ flow diagram is shown in Figure 1.

### Sample size

To determine the sample size, a similar study by Lin and colleagues (2002) was considered and a 20% rate of loss was assumed [21]. Therefore, 40 participants were needed for each group.

### Randomization

The participants were randomly allocated to the case or control group using simple random allocation.

### Intervention

The participants in the intervention group took part in 8 cardiac rehabilitation sessions. Rehabilitation sessions were held at Faghihi Hospital and consisted of fitness programs and educational programs lasting for 4 weeks. Two sessions were held each week lasting for 2 hours in the form of group education. The participants were trained regarding their use of drugs and diet, weight control (30 min), stress management, quitting smoking (45 min), light physical exercise, and relaxation (45 min). The contents of the program were developed by a research team consisting of a cardiologist and a heart surgeon. All patients were seen and examined by the research team before rehabilitation and then the questionnaires were given to patients by the researcher. In the rehabilitation center the patients were under the supervision of sport medicine experts too. The control group received the hospital’s routine postoperative care which was a cardiac rehabilitation pamphlet about their appropriate diet, physical activity and use of medication.

At the end of the intervention, educational booklets were given to the participants of the control group and they were referred to rehabilitation centers. If the patient in the control group was in need of education and rehabilitation during the study, he/she was given the appropriate education and excluded from the study.

### Instruments

Data were collected using a demographic information questionnaire, Spielberger’s Two-part Anxiety Scale (STAI) and Beck’s Depression Inventory (BDI).

Spielberger’s anxiety scale was used to determine the state and trait anxiety. The scale consisted of 40 questions and scores ranged from 40 points (no anxiety) to a maximum of 160 points. The first part of this two-part questionnaire consists of 20 questions focusing on the state anxiety, and the second part consists of 20 questions focusing on the trait anxiety. Each response is scored between 1 and 4, and total scores for each section (state and trait) are between 20–80. A score of 20–40 indicates mild anxiety, 41–60 moderate anxiety, and 61–80 severe anxiety [22]. The questionnaire’s validity and reliability have been reported by Hazavehei and Rymazewska [23,24]. The test-retest reliability (r = 0.97) and validity of the Persian version of the questionnaire was reported by Aghamohamadi et al. [25].

The Beck Depression Inventory is the most commonly used screening instrument for depression in the general population. This self-evaluation questionnaire is used to evaluate depression more extensively in psychiatric patients and to identify depression in other patients. The inventory contains 21 items and responses are scored from 1 to 3. Ghasemzadeh et al. (2005) reported high internal consistency of the Persian language version (Cronbach’s alpha = 0.87) and acceptable test-retest reliability (r = 0.74) in an Iranian sample [26]. Scores of 0–9 are considered normal, 10–16 indicate mild depression, 17 to 20 indicate moderate depression, 21–30 indicate severe depression, and scores > 30 indicate very severe depression.

The study was approved by the Ethics Committee of Shiraz University of Medical Sciences. The aim of the study was explained to each participant and their written informed consent was obtained. The participants were assured that all their personal information would remain confidential.

### Statistical analysis

The data were analyzed using SPSS software, version 15. We calculated relative and absolute frequencies, mean values and their standard deviation. Variables such as anxiety and depression were analyzed using multivariate repeated measurement and *t* tests, and demographic data were analyzed using frequencies and chi-square test. A p level of 0.05 was considered as statistically significant.

## Results

There were no statistical significant differences in demographic characteristics such as gender, age, educational level and occupation between the two groups. The participants’ mean ages were 59.2 years in the control group and 58.4 years in the intervention group (P = 0.67). In the control group 67.5% of the participants had completed primary school education and 32.5% had completed secondary school education. In the intervention group 72.5% had completed primary school and 27.5% had completed secondary school. Regarding their educational level, the two groups did not differ significantly (P = 0.31) (Table 1).

**Table 1:** Demographic characteristics of the participants by subgroups according to their frequency (%)

**Demographic Variables**		**Intervention group**	**Control Group**	**Total P value**
		(n = 40)	(n = 40)	
**Age (years)**	**40-50**	10(25)	7(17.5)	
	**51-60**	12(30)	15(37.5)	0.65
	**61-70**	18(45)	18(45)	
**Gender**	**Female**	13(32.5)	11(27.5)	
	**Male**	27(67.5)	29(72.5)	0.808
**Marital Status**	**Married**	35(87.5)	34(85)	0.5
	**Single**	5(12.5)	6(15)	
**Educational Level**	**Elementary**	29(72.5)	27(67.5)	0.404
	**Secondary or Higher**	11(27.5)	13(32.5)	

In the control group 30% of the participants were employed, 20% were unemployed, 25% were housewives and 25% were retired. In the intervention group 30% were employed, 17.5% were unemployed, 37.5% were housewives, and 15% were retired. The two groups did not differ significantly regarding this criterion (P = 0.40).

The mean depression and anxiety scores did not differ significantly between the two groups. Mean anxiety scores in the intervention group were 37, 31, and 28 before, immediately after and 2 months after rehabilitation, respectively. Mean anxiety scores in the control group were 38, 34 and 32. After the intervention, the level of anxiety in both case and control groups reduced but the difference between groups was not statistically significant (P = 0.079).

There was a statistically significant difference in depression scores between groups.

After the interventions the level of depression decreased significantly in both groups (P = 0.014). Our comparison of depression and anxiety scores before, immediately after, and 2 months after rehabilitation showed that time was an influential factor in reducing depression and anxiety. The mutual effect of time and group, using the multivariate repeated measures, showed that the interventions reduced depression (P = 0.001). Anxiety levels declined in both groups but the results indicated that the anxiety levels between groups was not significant (P = 0.079) (Table 2).

**Table 2:** Anxiety and depression scores before and after the intervention using repeated measurement tests

**Variable**	**Groups**	**Time (Mean ± SD)**			**P value**		
		**Before intervention**	**After intervention**	**2 months after intervention**	**Time**	**Group**	**Time/group**
**Anxiety**	**Case**	**37** ± 11.02	31 ± 6.29	28 ± 5.11	<0.001	0.079	0.007
	**Control**	38 ± 9.33	34 ± 7.76	32 ± 7.08			
**Depression**	**Case**	19.6 ± 4.54	15 ± 4.07	10.3.02	<0.001	0.014	0.001
	**Control**	19.5 ± 4.52	17.3.60	14 ± 3.28			

## Discussion

The present study explores the effect of cardiac rehabilitation on depression and anxiety among 80 patients undergoing CABG. We found that rehabilitation reduce depression and anxiety. The decreases in anxiety in both groups differed, but this difference was not statistically significant. There was a statistically significant difference in depression scores between groups.

In Hazavei et al’s study (2008) the effect of educational programs on depression was evaluated in 54 patients undergoing bypass surgery. The educational program consisted of relaxation, correct breathing exercises and appropriate physical exercises. The results showed that educational intervention was effective in the intervention group (P < 0.001) for reducing depression [23]. In another uncontrolled trial conducted in Iran, the researchers assessed the effect of progressive muscular relaxation training on postoperative anxiety and quality of life in patients who underwent CABG surgery. They found that after an interventional course consisting of exercise training, lifestyle education and muscular relaxation, anxiety levels reduced significantly, while quality of life scores increased compared to the usual care for the control group (exercise training and lifestyle education) [27]. In this study we did not mainly focus on relaxation, although relaxation was only a part of our educational cardiac rehabilitation intervention. In our study the mean anxiety level after the intervention reduced from 37 (before intervention) to 31 (after intervention) and 28 (2 months after intervention) but the difference between the three time points were not significant, in contrast to the previously mentioned study [27]. Although the difference between the three time-points was not significant, the reduction of mean anxiety score was lower than Hazavei et al’s study [23].

Lie et al. (2007) reported that 32% of the participants had anxiety before surgery in their study, which was reduced by 15% after the intervention [5]. They found that 6 weeks and 6 months after the intervention, the signs of anxiety and depression improved considerably in both case and control groups. However, no significant statistical difference was seen between the two groups, which is consistent with our results. The lack of significance may be related to the short duration of the interventions.

We believe that fear and worry about health and the future remains an important influence on the patients’ mental health even after rehabilitation. In this regard, Rymazewska and Kiejna (2003) noted that severe anxiety in patients before surgery declined after successful surgery and then increased gradually three months later [24]. Seki et al. (2003) observed a statistically significant difference in anxiety and depression 6 months after cardiac rehabilitation in patients suffering from stroke [28]. In a similar study by Dugmore et al. (1999) to determine the effect of cardiac rehabilitation on the quality of life in patients suffering from acute cardiac attacks, improvement was achieved only in the mental and psychological aspects of quality of life 4 months after the intervention [29]. These findings indicate the effect of cardiac rehabilitation on reducing depression in patients who have undergone CABG.

We found that two months after intervention, anxiety and depression scores reduced although the reduction was not significant for the anxiety scores between the groups. However, the insignificance could be due to the short duration or our interventional programs. One study on the effect of postoperative cardiac rehabilitation programs on anxiety as well as quality of life of patients after CABG, showed that anxiety levels significantly reduced in the patients, suggesting that longer interventional programs might be more beneficial [30].

Lie et al. (2007) assessed the effect of intervention at home on anxiety and depression in patients who underwent coronary artery bypass graft surgery in a psychoeducative intervention. The rate of depression decreased from 19% before the intervention to 13% after the intervention (5).

These findings are consistent with the effect of educational interventions in reducing depression in patients who underwent CABG, as found in the present study. Our findings are also consistent with those of Yoshida et al. (2002), who reported physical and psychological improvements after a 4-week period of stage-two cardiac rehabilitation in patients suffering from stroke [31].

In contrast, Mohammadi et al. (2007) assessed the effect of home-based cardiac rehabilitation on quality of life in 38 patients suffering from stroke. At base line the participants’ knowledge about disease management and quality of life were measured. Then the experimental group was instructed by multidisciplinary team about home based cardiac rehabilitation programs. The patients received information and support and guidance through phone calls and home visits. At the end of the three months follow-up, the patients’ knowledge and quality of life were measured again. She concluded that their intervention was not significant in social dimensions of quality of life and positive effect of home based CRP on QOL wasn’t supported [32]. Moreover, in the Birmingham rehabilitation uptake maximization (BRUM) study, in which the researchers compared home-based with center-based cardiac rehabilitation, no significant difference was found between the two types of cardiac rehabilitation [33]. Considering that our study was center-based, it seems that home or center based interventions might yield similar results.

### Limitation of the study

Although the mean anxiety was reduced in the intervention group the difference was not statistically significant between the groups. This may be because anxiety is a subjective, dynamic, and multidimentional concept and it’s improvement needs a larger sample with a longer period of time especially with the application of a comprehensive rehabilitation program.

## Conclusion

The results of this study provide evidence of the benefits of cardiac rehabilitation for depression and anxiety in patients undergoing CABG. We recommend that rehabilitation programs will be established to help prevent depression and anxiety among patients as well as to reduce healthcare costs and increase the patients’ quality of life. Our findings are potentially useful in planning nursing services for post-cardiac surgery rehabilitation, in nursing education, and in designing interdisciplinary rehabilitation programs.

## Competing interests

The authors declare that they have no competing interests.

## Authors’ contributions

FS, the main investigator, coordinated the research and wrote the first draft of the manuscript. AS was responsible for data collection and contributed to the data analysis. MJ assisted in the study design and liaised with the patients for the intervention. JK helped with patient referral after the diagnosis, provided feedback on the study design and manuscript, and liaised with the patients for the intervention. NZ did the data analysis and gave statistical advice. All the authors read and approved the final manuscript.

## Pre-publication history

The pre-publication history for this paper can be accessed here:

http://www.biomedcentral.com/1471-2261/12/40/prepub
